# Tracking Enterobacteria, microbiomes, and antibiotic resistance genes from waste to soil with repeated compost applications

**DOI:** 10.1371/journal.pone.0329200

**Published:** 2025-08-13

**Authors:** Syndia Sadikalay, Laura Cavé, Célia Ducat, Gwénola Mauriello, Mylène Berchel, Didier Boismoreau, Stéphanie Guyomard, Sylvie Nazaret, Antoine Talarmin, Séverine Ferdinand

**Affiliations:** 1 Unit of Health and Environment, Institut Pasteur de Guadeloupe, Les Abymes, France; 2 Research Group on « Multi-résistance environnementale et efflux bactérien », UMR Ecologie Microbienne CNRS/INRA-1418/Université de Lyon, Villeurbanne, France; 3 Association pour le maintien d’une agriculture paysanne, Petit-Bourg, France; Universidad San Francisco de Quito, ECUADOR

## Abstract

The dissemination of antibiotic resistant bacteria (ARB) and genes is one factor responsible for the increasing antibiotic resistance and the environment plays a role in resistance spread. Animal excreta can contribute to the contamination of the environment with ARBs and antibiotics and in some cases, environmental bacteria under antibiotic pressure may acquire antibiotic resistance genes (ARGs) from ARBs by horizontal gene transfer. In Guadeloupe, a French overseas department, organic amendments derived from human and animal waste are widely used in soil fertilization, but their contribution to antibiotic resistance remains unknown. The objective of this study was to evaluate the impact of composting animal and human raw waste and the repeated application of their derived-composts, on the fate of ARGs and antibiotic resistant Enterobacteria, for the first time, in tropical soils of Guadeloupe used for vegetable production. An unculturable approach was used to characterize the bacterial community composition and ARG content from raw waste to composts. A cultivable approach was used to enumerate Enterobacteria, and resistant isolates were further characterized phenotypically and genotypically. Based on this original approach, we demonstrated that the raw poultry droppings exhibited a depletion of Escherichia and Shigella populations during the composting treatment, which was corroborated by the results on the culturable resistant Enterobacteria. Significant differences in the abundance of ARGs were also observed, with some gene levels increasing or decreasing after composting. In addition, other bacterial genera potentially involved in the spread of antimicrobial resistance were identified. Taken together, these results demonstrate that successive applications of raw waste-derived-composts from green waste, sewage sludge, and poultry droppings reshape the Enterobacterial community and influences the abundance of ARGs, with some gene levels increasing or decreasing, in Guadeloupe’s tropical vegetable production soils.

## Introduction

Since the introduction of antibiotics, bacteria have found ways to resist antibacterial drugs. Generally, the description of acquired resistance to antibiotics closely follows the use of this antibiotic in medicine. Two main factors may explain the global increase of antibiotic resistance. The first is the selection pressure associated to the misuse of antibiotics in both human and veterinary medicine [1]; the second is related to the dissemination of antibiotic resistant bacteria (ARB). The environment plays a role in resistance dissemination as a reservoir of antibiotic resistance genes (ARGs) and mobile genetic elements (MGEs) particularly soil and water [2,3].

Avian and pig farms represent the largest consumers of antibiotics worldwide [4]. Furthermore, antibiotics are found in animal raw waste, with concentrations ranging from 40% to 90% for sulfonamides and tetracyclines administered as antibiotics. These antibiotics are partially metabolized and rapidly excreted, contributing to their presence in the environment [5]. It has been demonstrated that antibiotics released into the environment and animal excreta can contribute to the contamination of the environment with ARBs and antibiotics [6]. In some cases, environmental bacteria under antibiotic pressure may develop resistance through the selection or acquisition of ARGs from ARBs in their biotope by horizontal gene transfer.

The environmental dissemination of antibiotic resistance is a growing concern, particularly in agricultural settings where organic amendments derived from waste are commonly used. In many parts of the world, raw waste is regarded as a valuable, traditional and pervasive source of nutrients for crop production and an organic matter for the enhancement of soil quality. It has been demonstrated that soils directly fertilized by manure (chicken, pig, cattle) are a source of antibiotic-resistant bacteria [7,8]. The recycling of raw animal waste, or sewage sludge as fertilizers in crop production has been demonstrated to induce the spread of antimicrobial resistance in the environment [9]. However, the consequences for human health remain poorly understood. In Guadeloupe, a French overseas department located in the Caribbean, raw waste from human and animal sources are widely used in soil fertilization, particularly for market gardening crops. A large diversity of antimicrobial agents is used in veterinary medicine including ampicillin, sulfonamides and tetracyclines and their co-resistance serves as an indicator of multidrug resistance in *Escherichia coli* isolates from animals [10]. Vegetables are the most susceptible to the application of raw waste such as pig slurry, compost from poultry droppings manure, or horse feces manure or sewage sludge and the consumption of crops grown in soil fertilized by human and animal waste may result in increased exposure to ARBs and genetic determinants. In a geographical area characterized by a tropical climate, temperatures above 20°C, as well as high levels of humidity, these practices may be a source of high bacterial density development and favorable conditions for inter- and intra-species resistance genes and/or MGE exchange. While the impact of these amendments has been studied in temperate regions [11–14] their effect on tropical soils remains largely unknown. This study addresses this gap by investigating the impact of compost applications on Enterobacteria and ARGs in tropical soils.

The objective of this study was to evaluate the effects of composting on the bacterial community and antimicrobial resistance in different raw waste, namely horse and poultry droppings, green waste and sewage sludge. Additionally, the impact of repeated compost applications on the spread of ARGs and ARBs in soils grown with cucumber and sweet potatoes was investigated. The originality of this research lies in its combined approach, using both cultivable and unculturable methods to characterize bacterial community composition and ARGs content..

## Materials and methods

### Study area and experimental design

**Fig 1:**
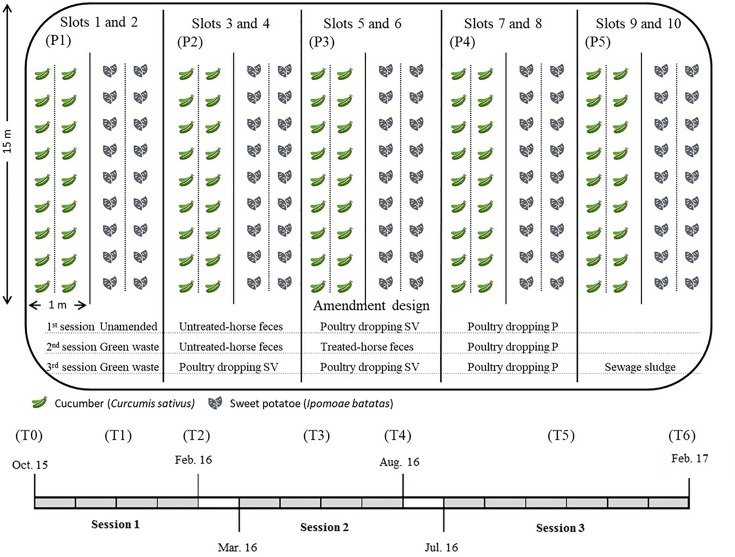
Amendment design. Experimental plot, amendment intake and temporal scale were designed as slots: P1: no raw waste/ green waste/ green waste – slots 1 and 2, P2: horse feces/ horse feces/ poultry dropping – slots 3 and 4, P3: poultry droppings/ horse feces/ poultry droppings – slots 5 and 6, P4: poultry droppings/ poultry droppings/ poultry droppings – slots 7 and 8, P5: no raw waste/ sewage sludge – slots 9 and 10; and time: T0: before the 1^st^ session, T1: after the first raw waste application in the middle of the 1^st^ session, T2: after plant harvest and before the second application at the end of the 1^st^ session, T3: after the second raw waste application in the middle of the 2^nd^ session, T4: after plant harvest and before the third application at the end of the 2^nd^ session, T5: after the third raw waste application in the middle of the 3^rd^ session, T6: after plant harvest at the end of the 3^rd^ session.

The experimental field plot, located in Petit-Bourg, was used for vegetable crop cultivation over two sessions. The plot was divided into 10 slots. Briefly, the study was carried out over two sessions with vegetable crops and sweet potatoes on amended plots. Two plots of unamended soil were used as control (Fig 1). The composts were applied at the average quantity typically used by farmers in three crop sessions of 5 months each. One month was allowed between each session. Detailed information on the plot characteristics, soil properties, and experimental design can be found in the Supporting information.

### Compost preparation and application

Composts were prepared from poultry droppings, horse feces, green waste, and sewage sludge using windrow composting. A minimum of 1 m^3^ of raw waste was used for all composts. Application rates were based on usual farming practices. Specific details on composting conditions (temperature, turning frequency, duration) and application rates are available in the Supporting information. The applications of the composts used per slot and session are detailed in Fig 1.

### Sampling and sample processing

Samples of raw waste, mature compost, soil, and vegetables were collected before compost application, during crop growth, and at harvest. Samples were processed by sieving and grinding. A full description of the sampling procedures, sample processing methods, and storage conditions is provided in the Supporting information.

### Bacterial isolation and microbiome composition

Bacterial isolation and count were performed using selective media. Species identification was performed using matrix-assisted laser desorption/ionization time-of-flight mass spectrometry (MALDI TOF). Detailed protocols for bacterial isolation, identification, and quantification are available in the Supporting information.

### Antimicrobial susceptibility testing

The antimicrobial susceptibility of all Enterobacteria strains isolated from raw waste, composts and vegetable samples was assessed using the disk diffusion technique on Mueller-Hinton agar, as recommended by EUCAST 2017 (http://www.eucast.org). The inhibition zones were measured using the Adagio™ automated system (Bio-Rad, Marnes-La-Coquette, France). Enterobacteria strains were classified as susceptible, intermediate, or resistant according to the guidelines of EUCAST. *E. coli* ATCC 25922 was used as the control strain.

### ARGs characterization and quantification

DNA from bacteria was extracted on Chelex-based resin, using an InstaGene™ Matrix kit (Biorad, California, USA). Total DNA from raw waste, compost, and soil samples on a silica membrane column using the NucleoSpin® soil kit (Macherey Nagel, Hoerdt, France). ARGs were identified by targeted standard PCR (*bla*_CTX-M_, *bla*_MOX_, *bla*_CMY_, *bla*_LAT_, *bla*_BIL_, *bla*_DHA_, *bla*_AAC_, *bla*_MIR_, *bla*_ACT_, and *bla*_FOX,_ *bla*_TEM_, *bla*_SHV_ and *sul*_,_ *qnr*B, *qnr*S genes) as previously described [15–19]. The amplified PCR products were sequenced by Eurofins (Ivry sur Seine, France) for further characterization and quantified by qPCR (*sul*1, *sul*2) using a Bio-Rad CFX96 real-time PCR instrument and ddPCR (*bla*_CTX-M_, *bla*_IMP-_ *qnr*A, *qnr*B *intI*1 and *intI*2) [20] using a QX100^TM^ Droplet Digital^TM^ PCR system. Detailed information on DNA extraction, qPCR conditions, and primer sequences (S2 Table) can be found in the Supporting information.

### Statistical analysis

The bacterial count results are presented as mean ± standard deviation (± sd). A one-way analysis of variance with a post-hoc Scheffe test was used to ascertain the differences in bacterial enumeration between raw waste and composts. The level of statistical significance was set at p < 0.05. The absolute gene levels between the different raw waste, composts, and soils were compared using the Wilcoxon-Mann-Whitney and Kruskal-Wallis tests. Gene amounts below the limit of detection (LOD) were replaced by the corresponding LOD values. All gene data were log-transformed prior to statistical analysis. *P*-values lower than 0.05 were considered statistically significant. The Operational Taxonomic Unit (OTU) composition in the composts, control and amended soils was subjected to non-metric multidimensional scaling (NMDS) analysis using Bray-Curtis dissimilarity distance.

## Results

### Bacterial community composition of raw waste and composts based on 16S metabarcoding

**Fig 2:**
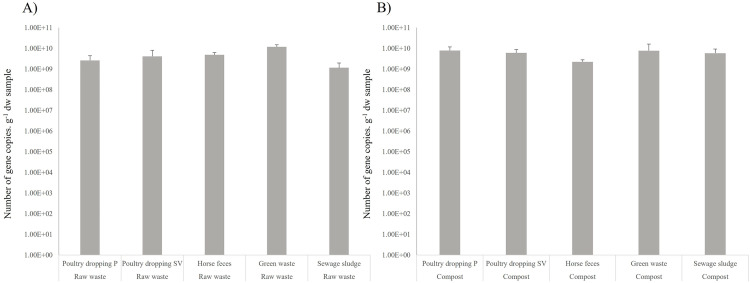
Bacterial community composition of raw waste and composts based on 16S metabarcoding. Abundance of 16S rDNA copies per g of sample dry weight in the raw waste (A) and their derived-composts **(B)**. Poultry dropping P corresponds to waste collected from the Pitaya farm and poultry dropping SV corresponds to waste collected from the Sita Verde company.

The amounts of 16S rDNA copies were of 1.2 (±0.81) x 10^9^ and 1.2 (±0.34) x 10^10^ per gram of dry weight sample (*i.e.* copies.g^-1^ sdw) in the raw waste and composts, respectively (Fig 2). Significant differences in the number of 16S rDNA copies between raw waste and their derived-composts were observed for poultry droppings, horse feces and sewage sludge (p < 0.05) (S1 Table), whereas no differences were observed between raw green waste and composted green waste.

The diversity of the bacterial communities from raw waste and composts was estimated using the richness (Chao1) and evenness (Shannon evenness) indices (S1 Fig). The Chao1 values ranged from 31868 (±1283) to 39720 (±6812) and from 17386 (±4830) to 28637 (±6918) and the Shannon values ranged from 8.8 (± 0.74) to 9.3 (±0.39) and from 8.8 (±1.4) to 10.3 (±0.24) for raw waste and composts, respectively. Variability was sometimes high due to differences in waste producers (e.g., Pitaya farm (P) *vs.* Sita Verde company (SV) for poultry droppings) or session, as waste batches differed over time.

The 16S rDNA metabarcoding data clearly showed differences between the different types of raw waste (S2A Fig) or between the different types of compost (S2B Fig). The communities from raw poultry droppings were the most diverse, whereas samples from raw sewage sludge showed the most similar communities.

A total of 1366 and 1410 genera were detected in the raw waste and composts, respectively. We looked for the 50 dominant genera in the different samples to assess whether the changes due to composting were related to the selection of similar indigenous populations present in the raw waste and then enriched by the composting treatment. We showed that Escherichia and Shigella are the most dominant in one out of the 6 poultry dropping samples analysed and rank between 32 and 45 in the others (S3 Table). These genera rank 12 and 15 out of 50 in the 2 horse feces samples. Escherichia and Shigella were no longer the dominant genera in composts from poultry droppings or horse feces as its mean abundance ranked 653 and 723, respectively when detected.

### Abundance of antibiotic resistant genes and integrons in raw waste and composts

A search for the prevalence of *β*-lactamase genes revealed the presence of *bla*_CTX-M_ and *bla*_IMP_. The former was detected only in raw poultry droppings at a concentration of about 10^6^ copies.g-^1^ sdw), while the latter was detected in all types of compost at levels of around 10^4^ copies.g-^1^ sdw. Notably, it was not detected in raw waste. The quinolone- resistant genes *qnr*A and *qnr*B were detected in the raw green waste at levels of approximately 5x10^5^ and 10^7^ copies.g-^1^ sdw) of sample, respectively.

**Fig 3:**
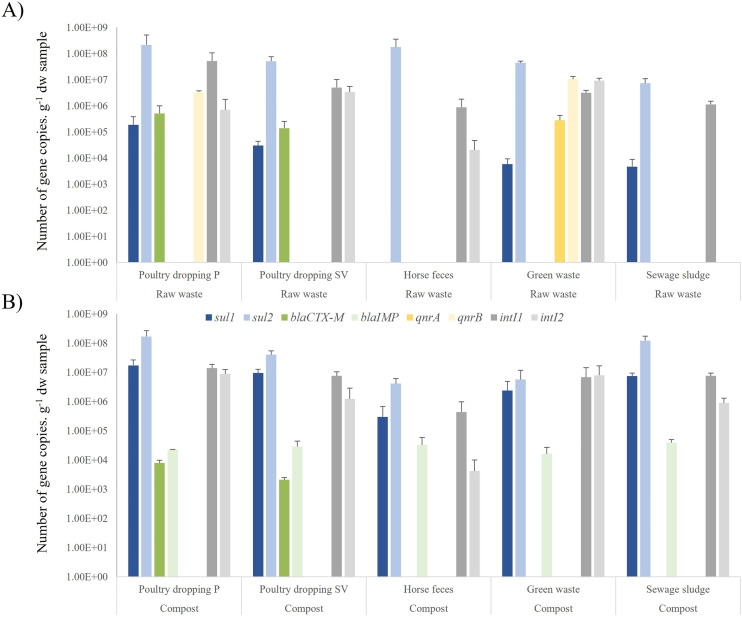
Absolute abundance of antibiotic resistance genes and integron-encoding genes in the raw waste (A) and their derived-composts (B). Poultry dropping P corresponds to waste collected from the Pitaya farm and poultry dropping SV corresponds to waste collected from the Sita Verde company.

The *sul*1 and *sul*2 genes were observed in all raw waste samples, except for the *sul*1 gene, which was either not detected or present below the detection threshold in raw horse feces (Fig 3). The results of the quantification indicated a higher abundance of the *sul*2 gene than the *sul*1 gene for all types of raw waste. The abundance of the *sul*1 gene varied from about 10^5^ to 10^7^ copies.g-^1^ sdw and that of *sul*2 from about 5x10^6^ to 10^9^ copies.g-^1^ sdw. The highest concentration of *sul* genes was observed in poultry droppings, followed by green waste and manure, and then sewage sludge.

Regardless of the ARG, a notable disparity in the concentration was observed between the unprocessed raw waste and their derived-composts (p < 0.01). With regards to *intI* genes, notable disparities were observed between the raw and composted sewage sludge samples (p < 0.001), with elevated levels of both genes detected in the composts. Nevertheless, the observed differences may be either an increase or a decrease in the number of ARG copies following the composting process. For example, the *bla*_IMP_ genes were never detected in the raw waste material but were detected in all compost samples. In contrast, *qnr* genes were detected in green waste but not in the composted green waste. The *sul*1 and *sul*2 genes were prevalent in all types of raw waste, with *sul*2 being more abundant overall, particularly in poultry droppings. The levels of these genes varied significantly among the raw waste sources. Regardless of the ARG, significant differences in abundance were observed between the raw waste and its derived-compost (p < 0.01).

### Abundance of antibiotic resistant genes (ARGs) and integrons in amended soils

**Fig 4:**
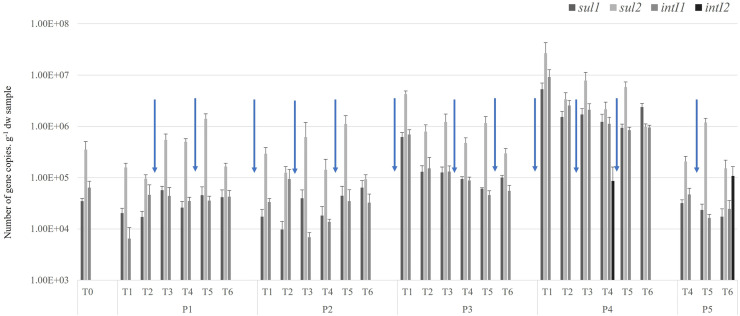
Absolute abundance of antibiotic resistance genes (*sul*1, *sul*2) and integron-encoding genes (*intI*1 and *intI*2) in soil over time after the various amendments. Arrows indicate the addition of composts.

The presence of *β*-lactamase and quinolone genes was looked for in control soils and in all amended soils that received a compost contaminated with these genes. Irrespective of the date of sampling, these genes were never detected, indicating that they are either absent or below the limit of detection. The *sul2* and *intI1* genes were detected in the control soils sampled at the beginning of the experiment (T0) at levels of about 10^6^ and 10^5^ copies.g-^1^ sdw, respectively. In contrast, the *sul*1 and *intI*2 genes were not detected before the start of the experiment (Fig 4). The application of compost resulted in the detection of the *sul*1 gene in the P3 at T1 (at a level of approximately 10^6^ copies.g-^1^ sdw) and in the P4 at all subsequent sampling points (from 10^6^ to 10^7^ copies.g-^1^ sdw). The detection of *intI*2 was also observed in P4 at T1, T4, T5, T6 and P5 at day T6 at levels ranging from approximately 10^4^ to 10^5^ copies.g-^1^ sdw.

The results showed that the prevalence of genes was influenced by the compost type and the target genes, with an increase in abundance observed in some cases (Fig 4). This seems to be closely related to the abundance of ARGs and integrons within the compost. For example, soils from plots P3 and P4, amended with poultry composts 2 or 3 times, showed the highest gene abundance, followed by plots amended with green waste or horse feces composts. The application of compost contaminated with ARGs increases the abundance of specific ARGs and integrons in the soil, with the most pronounced effect observed in plots amended with poultry composts.

### Abundance of cultivable resistant Enterobacteria in raw waste, composts and in amended soils

**Fig 5:**
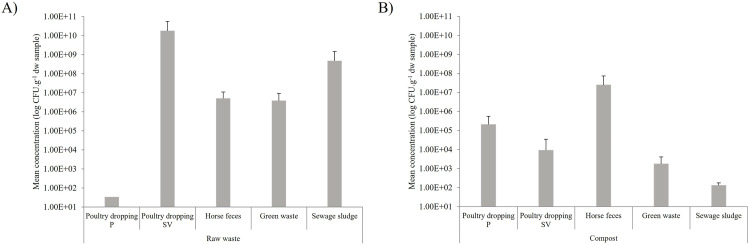
Ampicillin resistant Enterobacterial mean concentration in raw waste and their derived-composts (n = 41). **A)** Raw waste: Poultry dropping P (n = 4) and SV (n = 2), Horse feces (n = 5), Green waste (n = 2), Sewage sludge (n = 5). **B)** Composts: Poultry dropping P (n = 3) and SV (n = 3), Horse feces (n = 4), Green waste (n = 2), Sewage sludge (n = 2). Poultry dropping P corresponds to waste collected from the Pitaya farm and poultry dropping SV corresponds to waste collected from the Sita Verde company. CFU, colony forming unit. Mean concentrations are shown in CFU per gram of dry weight sample (CFU.g-^1^ sdw). Error bars represent the standard deviation of at least two independent experiments.

A total of 120 samples were collected during the 3 crop sessions. Twenty-four raw waste were collected prior to composting: sewage sludge (n = 5), feces from antibiotic treated- (n = 4) or untreated horses (n = 2), green waste (n = 3), poultry droppings from SV (n = 8) and P (n = 2). Twenty-one samples of compost and 30 soil samples were also collected and 45 vegetables: 21 sweet potatoes and 24 cucumbers were randomly sampled at harvest. The mean total resistant Enterobacteria enumerated on TTC + AMP media, classified as resistant (Amp^R^), decreased significantly from raw waste to composts (Fig 5). The highest abundance of Amp^R^ Enterobacteria was observed in poultry droppings (1.6x10^10^ CFU.g-^1^ sdw) followed by sewage sludge (4.7x10^8^ CFU.g-^1^ sdw), treated horse feces (7.3x10^6^ CFU.g-^1^ sdw), green waste (3.8x10^6^ CFU.g-^1^ sdw), and untreated horse feces (1.7x10^6^ CFU.g-^1^ sdw) (S3 Fig). Three out of 5 sewage sludge samples were below the detection limit (9,9x10^1^ CFU.g-^1^ sdw). Except for untreated horse feces, the abundance of Amp^R^ Enterobacteria after composting is lower than in corresponding raw waste, *i.e.,* 1.8x10^10^ CFU.g-^1^ sdw and 9.2x10^3^ CFU.g-^1^ sdw in raw *vs* composted SV poultry droppings, or 4.7x10^8^ CFU.g-^1^ sdw and 1.3x10^2^ CFU.g-^1^ sdw in raw *vs* composted sewage sludge. In the composts, the highest Amp^R^ Enterobacterial load was observed in untreated horse feces (3.3x10^7^ CFU.g-^1^ sdw). In the first session (T1 and T2), no Amp^R^ Enterobacteria were detected in soils regardless of the load in the compost (S4 Fig). The emergence of Amp^R^ Enterobacteria was quantified in soils. Irrespective of the amendment type, successive applications resulted in the detection of Enterobacteria in soils, but at a lower abundance than in composted raw waste (Fig 4 and S4 Fig), 1.8x10^4^ CFU.g-^1^ sdw *vs* 6.2x10^6^ CFU.g-^1^ sdw respectively.

### Species diversity of cultivable resistant Enterobacteria in raw waste, composts, soils and vegetables

Potentially resistant Enterobacterial species were quantified by calculating the ratio of bacteria per species on Lactose-triphenyl tetrazolium chloride-agar with Tergitol-7 (TTC) with and without antibiotic agents (ampicillin, imipenem, ciprofloxacin, ceftriaxone). Enterobacteria enumerated on these selective media were classified as resistant. The mean concentration of resistant Enterobacteria per species in composts, soils, and vegetables according to slots (P1 to P5) and time sessions (T0 to T6) is shown in S5 Fig.

Despite the contribution of resistant *E. coli via* the second application of green waste compost (S2A Fig) or SV poultry dropping compost (S2B Fig), these were never found in soils; other Enterobacteria species, *E. cloacae* and *E. asburiae* appeared (S2A Fig). Similarly, after the second application of P poultry dropping compost (S2D Fig), resistant *K. pneumoniae* (81%), *E. aerogenes* (10%) and *E. asburiae* (9%) were detected in soil 1 month after harvest. These bacteria were undetectable at the end of the experiment (S2D Fig). Untreated horse feces and SV poultry dropping composts are sources of resistant bacteria (S2B Fig), but the former was richer in resistant Enterobacteria (3.3x10^7^ CFU.g-^1^ sdw *vs* 9.2x10^3^ CFU.g-^1^ sdw) and had a greater species diversity: *E. cloacae*, *E aerogenes*, *Enterobacter* sp., *Citrobacter* sp., *C. koseri*, *S. marcescens vs* 3 species for SV poultry droppings: *E. coli*, *E. cloacae*, *P. stuartii*. Repeated applications of these composts resulted in the persistence of *E. cloacae* in the soil 1 month after harvest at the end of the experiment (S2B and C Fig). Sewage sludge composts did not appear to be a source of resistant Enterobacteria (1.3x10^2^ CFU.g-^1^ sdw). However, the effect of this raw waste was only investigated once at the end of the project. The concentration of resistant Enterobacteria in compost derived from treated horse feces was below the detection limit of this study (6.5x10^1^ CFU.g-^1^ sdw). Despite being prevalent in raw waste, no resistant *E. coli* were detected in compost or vegetables. Resistant *E. coli* levels were also reduced in soils. In contrast, resistant *K. pneumoniae* was found only in soils, indicating a shift in the species diversity of resistant Enterobacteria at different stages from raw waste to vegetables at harvest.

Few resistant Enterobacteria were detected on sweet potatoes or cucumbers grown on amended plots (10^2^ to 10^3^ CFU.g-^1^ sdw). Four species not detected previously in raw waste or soil were recovered from vegetables, *Enterobacter cowanii*, *Enterobacter hirae*, *Pantoea dispersa*, *Pantoea agglomerans*. Other species, such as *E. aerogenes*, which were not detected in raw waste and were specifically from soils, were detected at lower levels in cucumbers and sweet potatoes. The emergence of different resistant Enterobacterial species was observed in cucumber and sweet potatoes crops, even when the same raw waste was applied. For example, *E. coli* and *E. hermannii* were detected in cucumbers and *E. aerogenes* in sweet potatoes on the same slots amended green waste; *E. cowanii* was detected in cucumbers while *E. aerogenes* and *E. hirae* were detected in sweet potatoes on the same slots amended with P poultry droppings. A diversity of resistant Enterobacterial species was found on vegetables grown on amended soils, including some species not previously detected in raw waste or soil, suggesting that vegetable production can select for distinct populations of resistant bacteria.

### Antibiotic resistance profile and ARG content among cultivable resistant Enterobacteria

A total of 216 antibiotic-resistant Enterobacteria were isolated. Among them, 195 (90.3%) were *E. coli* from raw waste (131 isolates from treated horse feces, 47 from SV poultry droppings, 17 from sewage sludge). High rates of resistance to ampicillin and ticarcillin (81.5% and 81.9% of resistant strains respectively), to trimethoprim-sulfamethoxazole (81.9%) and quinolones (70.8% resistance to nalidixic acid and 67.1% to ciprofloxacin) were observed. Lower resistance rates were observed for cefalexin (26.9%, 1^st^ generation), cefotaxime (26.9%, 3^rd^ generation), and ceftazidime (26.4%, 3^rd^ generation). Resistance to the combination amoxicillin + clavulanic acid was detected in 14.4% of the isolates.

**Fig 6:**
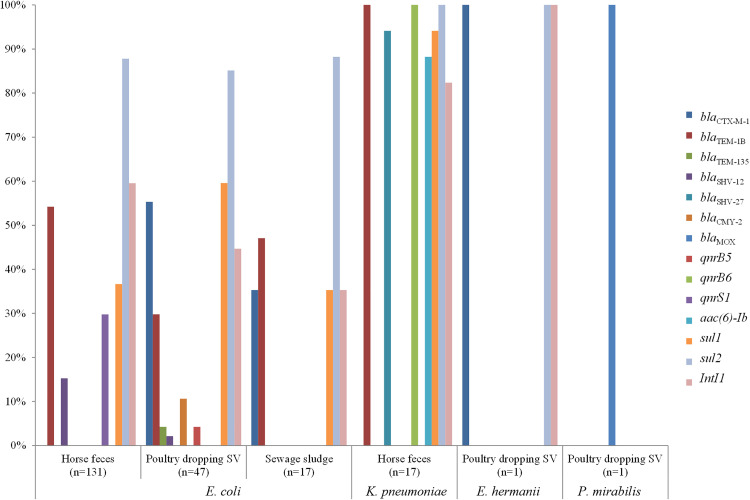
Proportion of resistance genes by species and raw waste (n = 214). *β*-lactamase-encoding genes: *bla*_CTX-M_, *bla*_TEM_, *bla*_SHV_, *bla*_CMY,_ *bla*_MOX._ Quinolone-encoding genes: *qnr,* aminoglycoside-modifying-encoding gene: **aac [*6*]-Ib.** Sulfonamides-encoding genes *sul.* Class I integrase-encodig gene: *intI.* Poultry dropping P corresponds to waste collected from Pitaya farm and poultry dropping SV corresponds to waste collected from Sita Verde company. While 216 resistant Enterobacteria strains were isolated, the graph displays only 214. One *E. coli* strain (found in compost from untreated horse manure) and one *P. stuartii* strain (not screened for resistance genes) are excluded from this figure.

The distribution of resistance genes carried by resistant Enterobacteria showed a predominance of the *sul*2 gene in raw waste, representing more than 80% of isolates in each waste. The *bla*_CTX-M-1_ gene, mainly carried by *E. coli*, was predominant in SV poultry droppings (27/49, 55.1%) and sewage sludge (6/17, 35.3%, p < 0.0001), while the *bla*_TEM-1B_ and *bla*_SHV-12/27_ genes, shared by *E. coli* and *K. pneumoniae*, were more prevalent in treated-horse feces (88/148, 59.5% and 36/148, 24.3% respectively, p < 0.003). Treated horse feces were also the main source of *sul* (135/148, 91.2%), *qnr* and **aac*(6)*-Ib** resistance genes (60/148, 40.5% and 15/69, 21.7% respectively), and *intI*1 integrase coding gene (92/148, 62.2%) (Fig 6). High rates of resistance to common antibiotics such as ampicillin, ticarcillin, trimethoprim-sulfamethoxazole, and quinolones were found in antibiotic-resistant Enterobacteria isolated from raw waste, with a predominant distribution of the *sul*2 gene in these isolates.

## Discussion

The present study sought to evaluate the impact of composting on animal and human raw waste and the repeated application of their derived-composts, on the fate of antibiotic resistance genes and antibiotic resistant Enterobacteria in tropical soils of Guadeloupe used for vegetable production. The unculturable approach demonstrated that the raw poultry droppings exhibited a depletion of Escherichia and Shigella populations during the composting treatment, which was corroborated by the results on the culturable resistant Enterobacteria. Furthermore, significant differences in the abundance of ARGs were observed, with some gene levels increasing or decreasing after composting. Additionally, other potential bacterial genera involved in the spread of antimicrobial resistance were identified. Taken together, these findings confirm that composting is an effective microbial remediation process, irrespective of the inputs employed (green waste, sewage sludge, poultry droppings) [7,8].

The finding of our study corroborate earlier research, which has demonstrated that composting significantly reduces the initial amount of pathogenic bacteria, antibiotics and ARGs, particularly under thermophilic conditions which facilitate thermophilic activity [9,11,12]. This observation is corroborated by the reduction in Escherichia and Shigella populations, as well as culturable resistant Enterobacteria in the composted samples. However, while a general decrease in ARG abundance was observed after composting, certain ARGs persisted or even increased. This is consistent with previous studies that have reported ARG persistence despite the absence of detectable bacteria [13]. This highlights the complexity of ARG dynamics, which can be influenced not only by microbial viability but also by the stability of genetic elements such as MGEs [21].

Although composted raw waste was less loaded with resistant Enterobacteria than the corresponding raw amendments, we showed that composts are reservoirs of resistant Enterobacteria leading to their detection in soils after successive applications. The detection of resistant Enterobacteria in soil post-application is in line with previous research demonstrating that composts can act as reservoirs for AMR bacteria, especially under repeated use scenarios [14]. Furthermore, the effect of compost on bacterial communities may vary depending on factors such as compost quality, application frequency, and soil type [22]. In our study, resistant Enterobacteria were detected in soil, but they differed in terms of species diversity to those present in the original raw waste. Initially, the soil contained indigenous species such as *E. coli*, *K. pneumoniae*, *E. cloacae*. While compost applications may introduce other species like *P. stuartii*, *P. dispersa*, *C. amalonaticus*, these are not detected in cultivated soils. In these environments, endogenous Enterobacteria, for instance, i.e., *E. aerogenes*, *E. asburiae* as identified in our study, are more likely to be detected, probably due to their better adaptation than exogenous populations in the amendments.

We demonstrated that the use of successive amendments from different sources influenced the application load and the species diversity of resistant Enterobacteria. The unexpected lowest level of resistant Enterobacteria in compost of treated horse feces compared to untreated may be related to the lowest Enterobacterial population diversity, probably more homogeneous and susceptible to temperature increase during composting than in untreated horse feces, where different Enterobacterial species as *Klebsiella pneumoniae*, *Enterobacter* sp. *Citrobacter* sp. *Serratia rubidae* were isolated. This dynamic may prevent the accumulation of resistance and maintain the equilibrium of the biotope, avoiding the contaminants in the soil and subsequent uptake by plants. Our results also showed that the enrichment of soils with ARGs after successive applications seems to be closely related to the abundance of ARGs and MGEs in the compost and influenced by the application rate, with consequences for species diversity of resistant Enterobacteria. These findings are consistent with previous results showing that the microbial community and MGEs are also two factors that have a major influence on ARGs [21]. Furthermore, different microbiome compositions influence the profile and abundance of ARGs present, thereby shaping the soil resistome [23]. Yet, our study uniquely contributes to the literature by tracing the fate of resistance along the continuum from successive compost applications to soils and vegetables, revealing a disconnect in species continuity and suggesting environmental filtering and host-specific colonization as important determinants of bacterial persistence. In addition, our observation that the diversity of resistant Enterobacteria varies with compost source and application history builds on work showing that compost quality and microbial community composition strongly influence the prevalence of ARGs [22].

In our study, the application of composted raw waste such as poultry dropping compost, horse feces compost and sewage sludge compost did not appear to contribute to the spread of antimicrobial resistance in crop production *via* Enterobacteria. Although manure application is an important pathway for ARGs to enter agricultural soils, this finding supports the suggestion that manure-derived ARGs may have a low risk of transfer to root vegetable endophytes [24]. These suggestions were supported by the results of the resistance gene screening. Indeed, *E. coli* isolates, resistant to quinolones and/or β-lactamins, which are more common in animals than in human waste, were not detected in composts. In raw waste, the resistance genes detected in ESBL *E. coli*, *K. pneumoniae*, *P. stuartii*, *E. hermannii* differed according to the origin of the waste. In poultry droppings and sewage sludge, *bla*_CTX-M-1_ was the only gene detected, whereas in treated horse feces, *bla*_SHV-12_ was the most predominant gene [25]. This *bla*_SHV-12_ variant has been found sporadically in companion animals [26,27], in food-producing animals such as poultry, pigs, cattle [26–31], and in wild fauna [32], but to the best of our knowledge this *bla*_SHV-12_ variant was recently found for the first time in horse feces [25]. Finally, our identification of resistance genes such as *bla*_SHV-12_ in treated horse feces adds a novel dimension to the known distribution of ESBL genes across animal waste types highlighting the need for monitoring underrepresented sources [17]. Other studies conducted by our research team on the genotypic characterization of human *E. coli* (found in patients with UTI, or in water upstream and downstream of sewage sludge) have shown that ESBL *E. coli* were most often carriers of the *bla*_CTX-M15_ gene [33,34] and in rats the *bla*_TEM-52_ gene was frequently found [35]. Taken together, these observations could indicate a different distribution of resistance genes between soils and cultivated crops. The results highlight that while composting is effective in reducing the load of resistant Enterobacteria compared to raw waste, successive applications of compost avoid disturbing the soil biotope balance and spreading resistance. The present study corroborates the established benefits of composting in reducing AMR risk. Furthermore, it deepens current understanding by illustrating how compost source, application frequency, and microbial community structure interact to shape AMR outcomes across the soil-plant continuum.

The findings of this study highlight the value of composting as a viable strategy for significantly reducing the presence of resistant Enterobacteria and the load of antibiotic resistant bacteria and genes into cultivated soils, compared to raw waste. The adoption of composting as a standard pre-treatment for organic amendments has been demonstrated to promote safer and more sustainable agricultural practices. However, the persistence of certain ARGs as well as the detection of resistant bacteria in soils and, to a lesser extent, in crops, highlight the need for careful monitoring and management of compost application, especially with regard to its frequency and origin. From a public health perspective to minimiseing the transfer of antimicrobial resistance from the agricultural environment to the human population is important. Although our results suggest that composted amendments pose a lower risk than raw waste, they are not risk-free, as resistant bacteria can still enter the food chain via soil or vegetables. Therefore, the implementation of strict guidelines for compost production, application and crop handling could serve as a preventative measure against the environmental spread of antimicrobial resistance and ultimately contribute to efforts to curb the global AMR crisis.

## Conclusions

Composting and the successive application of raw waste derived-composts, in tropical soils of Guadeloupe used for vegetable production, reshape the bacterial community and influence the abundance of ARGs. Our findings highlight composting appears as an effective strategy for reducing the prevalence of specific antibiotic resistance genes, modifying the microbiome, and potentially limiting the spread of antibiotic resistance genes, in line with the broader goal of sustainable agriculture. However, further research is warranted to investigate the long-term effects of compost application on soil resistomes, to track the horizontal transfer of ARGs within the soil microbiome, and to assess the efficacy of different composting methods in eliminating a wider range of ARGs. Exploring the impact of compost origin (e.g., animal vs. plant) and application frequency on the persistence and spread of ARGs would also provide valuable insights for refining best management practices and mitigating potential risks to public health.

## Supporting information

S1 Table p-values of Wilcoxon tests comparing the abundance of antibiotic resistance and mobility genes between the raw waste and their derived-composts. (DOCX)

S2 Table qPCR and ddPCR primers and probes. (DOCX)

S3 Table Proportion of the reads corresponding to the 50 dominant genus in the bacterial communities of poultry dropping and horse feces. Escherichia and Shigella genera are labelled in bold.
(DOCX)

S1 Fig Diversity estimation of bacterial communities based on 16S metabarcoding. Box-plots of the Chao (A and B) and Shannon indexes (C and D) for the raw waste and their derived-composts.
(TIF)

S2 Fig Bacterial community composition of the raw waste (A) and their derived-composts (B). Bray–Curtis similarity coefficients were calculated from relative OTU abundances of bacterial communities and plotted on a nonmetric multidimensional scaling (NMDS) graph.
(TIF)

S3 Fig Resistant enterobacterial mean concentration in raw waste and their derived-composts according to horse antimicrobial treatment status (n = 9). A) Raw waste: Untreated horse feces (n = 2), Antibiotic-treated horse feces (n = 3) B) Composts: Untreated horse feces (n = 3), Antibiotic-treated horse feces (n = 1). ATB: antibiotic. CFU, colony forming unit. Mean concentrations are shown in CFU per gram of dry weight sample (CFU.g^-1^ sdw). Error bars represent the standard deviation of at least two independent experiments.
(TIF)

S4 Fig Resistant Enterobacteria abundance in soil after successive applications of amendments during crop production cycles. Six plots (P1 to P6) were loaded at different times session during the crop production periode (T0 to T6), according to the following sheme; P1: no raw waste/ green waste/ green waste – slots 1 and 2, P2: horse feces/ horse feces/ poultry dropping – slots 3 and 4, P3: poultry droppings/ horse feces/ poultry droppings – slots 5 and 6, P4: poultry droppings/ poultry droppings/ poultry droppings – slots 7 and 8, P5: no raw waste/ sewage sludge – slots 9 and 10; and time: T0: before the 1^st^ session, T1: after the first raw waste application in the middle of the 1^st^ session, T2: after plant harvest and before the second application at the end of the 1^st^ session, T3: after the second raw waste application in the middle of the 2^nd^ session, T4: after plant harvest and before the third application at the end of the 2^nd^ session, T5: after the third raw waste application in the middle of the 3^rd^ session, T6: after plant harvest at the end of the 3^rd^ session.
(TIF)

S5 Fig Mean concentration of resistant enterobacteria by species in composts (C), soils (T0 to T6) and vegetables (CC and SP) after successive applications of amendments (P1 to P5). A) P1 successive application of no raw waste/ green waste/ green waste, B) P2 successive application of horse feces/ horse feces/ poultry dropping, C) P3 successive application of: poultry droppings/ horse feces/ poultry droppings, D) P4 successive application of poultry droppings/ poultry droppings/ poultry droppings, E) P5 successive application of no raw waste/ sewage sludge, F) Mean concentration of resistant enterobacteria by species in vegetables (CC and SP) after successive applications of amendments (P1 to P5). T0: before the 1^st^ session, T1: after the first raw waste application in the middle of the 1^st^ session, T2: after plant harvest and before the second application at the end of the 1^st^ session, T3: after the second raw waste application in the middle of the 2^nd^ session, T4: after plant harvest and before the third application at the end of the 2^nd^ session, T5: after the third raw waste application in the middle of the 3^rd^ session, T6: after plant harvest at the end of the 3^rd^ session. No plot indicates that no resistant enterobacteria were detected, except for slots 9–10 (P5) where sewage sludge compost was applied only in the 3^rd^ session. CFU: colony forming unit, C: compost, CC, cucumber; SP, sweet potatoes, NA: not applicable.
(TIF)

S1 File Additional supplementary files are available in the Supporting information. These include detailed descriptions of materials and methods (such as plot characteristics, soil properties, experimental design, composting conditions, application rates, sampling procedures, bacterial analysis protocols, and molecular methods including DNA extraction, qPCR conditions, and primer sequences), as well as supplementary tables (S1–S3) and figures (S1–S5) illustrating the results.
(DOCX)
