# Correction: Tracking Enterobacteria, microbiomes, and antibiotic resistance genes from waste to soil with repeated compost applications

**DOI:** 10.1371/journal.pone.0337209

**Published:** 2025-11-18

**Authors:** Syndia Sadikalay, Laura Cavé, Célia Ducat, Gwénola Mauriello, Mylène Berchel, Didier Boismoreau, Stéphanie Guyomard, Sylvie Nazaret, Antoine Talarmin, Séverine Ferdinand

S1 File was uploaded incorrectly. Please see the correct S1 File here.

## Supporting information

S1 File Additional supplementary files are available in the Supporting information. These include detailed descriptions of materials and methods (such as plot characteristics, soil properties, experimental design, composting conditions, application rates, sampling procedures, bacterial analysis protocols, and molecular methods including DNA extraction, qPCR conditions, and primer sequences), as well as supplementary tables (S1–S3) and figures (S1–S5) illustrating the results.
(PDF)
