# Supplementary material for: Tracking Enterobacteria, microbiomes, and antibiotic resistance genes from waste to soil with repeated compost applications
Source: PLoS One. 2025 Aug 13;20(8):e0329200. doi: 10.1371/journal.pone.0329200 (PMC12349694; doi:10.1371/journal.pone.0329200)
Supplement: S1 Table — (DOCX) [file pone.0329200.s001.docx]

|  | **All samples** | **Poultry droppings** | **Horse feces** | **Sewage sludge** | **Green waste** |
| --- | --- | --- | --- | --- | --- |
| ***sul*1** | 1.0E-10 | 7.4E-07 | 0.0074 | 0.0009 | 0.0095 |
| ***sul*2** | 0.2600 | 0.0052 | 0.0041 | 0.0001 | 0.0095 |
| ***qnr*A** | 0.0420 | NA | NA | NA | 0.0057 |
| ***qnr*B** | 0.0420 | NA | NA | NA | 0.0057 |
| ***bla*_CTX-M_** | 0.0002 | 1.02E-05 | NA | NA | NA |
| ***bla*_IMP_** | 2.1E-13 | 1.01E-05 | 0.0074 | 7.2E-05 | 0.0110 |
| ***intI*1** | 0.1700 | 0.1600 | 0.2100 | 0.0001 | 1,0000 |
| ***int*2** | 0.1500 | 0.1700 | 0.3900 | 7.5E-05 | 1,0000 |
| **16S rDNA** | 0.0410 | 0.0056 | 0.0040 | 0.0004 | 0.6100 |
