# Supplementary material for: Tracking Enterobacteria, microbiomes, and antibiotic resistance genes from waste to soil with repeated compost applications
Source: PLoS One. 2025 Aug 13;20(8):e0329200. doi: 10.1371/journal.pone.0329200 (PMC12349694; doi:10.1371/journal.pone.0329200)
Supplement: S2 Table — (DOCX) [file pone.0329200.s002.docx]

| Gene | Sequence |  | Reference |
| --- | --- | --- | --- |
| *bla*_CTX-M1_ | blaCTXM1_F | CAGCTGGGAGACGAAACGTT |  |
|  | blaCTXM1_R | CCGGAATGGCGGTGTTTA | Hartmann et al. 2012 |
|  | blaCTXM1_probe | FAM- CGTCTCGACCGTACCGAGCCGAC-BHQ1 |  |
| *bla*_CTX-M9_ | blaCTXM9_F | GAGGCGTGACGGCTTTTG |  |
|  | blaCTXM9_R | CGTAGGTTCAGTGCGATCCA | Hartmann et al. 2012 |
|  | blaCTXM9_probe | FAM- CGATCGGCGATGAGACGTTTCGT-BHQ1 |  |
| *bla*_IMP_ | IMP-qF | GGCGGAATAGAGTGGCTTAATTCTC |  |
|  | IMP-qR1 | GAATTTTTAGCTTGTACTTTACCGTCTTT |  |
|  | IMP-qR2 | ATTTTTAGCTTGTACCTTACCGTATT | Van der Zee et al. 2014 |
|  | IMP-qR3 | TTTGTAGCTTGCACCTTATTGTCTTT |  |
|  | IMP-probe | FAM- ATGCATCTGAATTAAC-BHQ1 |  |
| *sul*1 | qSUL1F653 | CCGTTGGCCTTCCTGTAAAG | Pei et al. 2006 |
|  | qSUL1R719 | TTGCCGATCGCGTGAAGT |  |
|  | SUL1-probe | FAM-CAGCGAGCCTTGCGGCGG-BHQ1 |  |
| *sul*2 | qSUL2F595 | CGGCTGCGCTTCGATT | Heuer et al. 2008 |
|  | qSUL2R654 | CGCGCGCAGAAAGGATT |  |
|  | SUL2-probe | FAM-CGGTGCTTCTGTCTGTTTCGCGC-BHQ1 |  |
| *qnr*A | qnrA_RT_F | CAGTTTCGAGGATTGCAGTT | Vien et al. 2012 |
|  | qnrA_RT_R | CCTGAACTCTATGCCAAAGC, |  |
|  | qnrA_probe | FAM-AAGGGTGYCACTTCAGCTATGCC-BHQ1 |  |
| *qnr*B | qnrB_RT_F | CAGATTTYCGCGGCGCAAG | Vien et al. 2012 |
|  | qnrB_RT_R | TTCCCACAGCTCRCAYTTTTC |  |
|  | qnrB_probe | FAM-CGCACCTGGTTTTGYAGYGCMTATATCAC-BHQ1 |  |
| *int*1 | intI1-LC1 | GCCTTGATGTTACCCGAGAG | Barraud et al. 2010 |
|  | intI1-LC5 | GATCGGTCGAATGCGTGT |  |
|  | intI1-probe | HEX-ATTCCTGGCCGTGGTTCTGGGTTTT-BHQ1 |  |
| *int*2 | intI2-LC2 | TGCTTTTCCCACCCTTACC | Barraud et al. 2010 |
|  | intI2-LC3 | GACGGCTACCCTCTGTTATCTC |  |
|  | intI2-probe | FAM-TGGATACTCGCAACCAAGTTATTTTTACGCTG-BHQ1 |  |
| 16S rDNA | BAC338-F | ACTCCTACGGGAGGCAG | Knapp et al. 2010 |
|  | BAC805-R | GACTACCAGGGTATCTAATCC |  |
|  | BAC516-F | FAM-TGCCAGCAGCCGCGGTAATAC-BHQ1 |  |
