# Supplementary material for: Tracking Enterobacteria, microbiomes, and antibiotic resistance genes from waste to soil with repeated compost applications
Source: PLoS One. 2025 Aug 13;20(8):e0329200. doi: 10.1371/journal.pone.0329200 (PMC12349694; doi:10.1371/journal.pone.0329200)
Supplement: S3 Table — Escherichia and Shigella genera are labelled in bold. (DOCX) [file pone.0329200.s003.docx]

| Rank | Poultry dropping 1 | | Poultry dropping 2 | | Poultry dropping 3 | | Poultry dropping 4 | | Poultry dropping 5 | | Poultry dropping 6 | | Horse feces 1 | | Horse feces 2 | |
| --- | --- | --- | --- | --- | --- | --- | --- | --- | --- | --- | --- | --- | --- | --- | --- | --- |
|  | Genus | Proportion of reads | Genus | Proportion of reads | Genus | Proportion of reads | Genus | Proportion of reads | Genus | Proportion of reads | Genus | Proportion of reads | Genre | Proportion of reads | Genre | Proportion of reads |
| 1 | Gracilibacillus | 0.33416060 | **Escherichia_Shigella** | 0.48394327 | Jeotgalicoccus | 0.21587033 | Flavobacterium | 0.36836786 | Pseudomonas | 0.07679178 | Cloacibacillus | 0.05871861 | Verrucomicrobium | 0.09215019 | Solibacillus | 0.16777850 |
| 2 | Bacillus | 0.12139313 | Faecalibacterium | 0.07617128 | Bacillus | 0.18724793 | Pseudomonas | 0.09246042 | Solibacillus | 0.05259077 | Phascolarctobacterium | 0.05390943 | Luteolibacter | 0.03893889 | Acinetobacter | 0.07570591 |
| 3 | Salinicoccus | 0.07989452 | unclassified | 0.03048401 | Paucisalibacillus | 0.07198264 | Serpens | 0.04297239 | Serpens | 0.04623023 | Kurthia | 0.04219672 | Rummeliibacillus | 0.02420106 | Alkanindiges | 0.05297860 |
| 4 | Oceanobacillus | 0.06104561 | Tannerella | 0.01667701 | Cerasibacillus | 0.04444618 | Persicivirga | 0.03482777 | unclassified | 0.04157615 | Faecalibacterium | 0.04064537 | Aspromonas | 0.02319268 | unclassified | 0.04173131 |
| 5 | Halalkalibacillus | 0.05243562 | Robinsoniella | 0.01473783 | Oceanobacillus | 0.03459511 | unclassified | 0.03475023 | Anaerosporobacter | 0.04095564 | Pseudomonas | 0.03862858 | Serpens | 0.02280485 | Lysinibacillus | 0.03420727 |
| 6 | Solibacillus | 0.02970835 | Acetivibrio | 0.01202296 | Halalkalibacillus | 0.02660565 | Amphibacillus | 0.03040646 | Azorhizophilus | 0.04080050 | Megamonas | 0.03699969 | Stakelama | 0.02241701 | Anaerophaga | 0.03009619 |
| 7 | Virgibacillus | 0.02823457 | Wautersiella | 0.01101459 | Ornithinibacillus | 0.02365809 | Azorhizophilus | 0.02016754 | Parasporobacterium | 0.02381322 | Phocaeicola | 0.03343160 | Pseudoxanthomonas | 0.02233944 | Kurthia | 0.02598511 |
| 8 | Falsibacillus | 0.02606268 | Subdoligranulum | 0.01085945 | Allobacillus | 0.02295998 | Azomonas | 0.01706485 | Megamonas | 0.02280484 | Serpens | 0.03304376 | Anaerobacter | 0.01977971 | 3_genus_incertae_sedis | 0.02373565 |
| 9 | Sediminibacillus | 0.02295998 | Verrucomicrobium | 0.01078188 | Gracilibacillus | 0.01807323 | Bacillus | 0.01659944 | Cloacibacillus | 0.02233944 | Solibacillus | 0.03063916 | unclassified | 0.01962456 | Anaerosporobacter | 0.02288241 |
| 10 | Natronobacillus | 0.02288241 | Cellulosilyticum | 0.01070432 | Falsibacillus | 0.01574620 | Natronobacillus | 0.01644431 | Stenotrophomonas | 0.01923673 | Azorhizophilus | 0.02955322 | Faecalibacterium | 0.01559107 | Robinsoniella | 0.02280485 |
| 11 | Paucisalibacillus | 0.01721999 | Anaerosporobacter | 0.01000621 | Atopostipes | 0.01535837 | Petrimonas | 0.01442755 | Phascolarctobacterium | 0.01877133 | Parasporobacterium | 0.02148620 | Blastomonas | 0.01489296 | Caryophanon | 0.02202917 |
| 12 | Alkalibacterium | 0.01497053 | Gibbsiella | 0.00907540 | Natronobacillus | 0.01481539 | Tissierella | 0.01388458 | Cellvibrio | 0.01776293 | unclassified | 0.02148619 | Millisia | 0.01481539 | **Escherichia_Shigella** | 0.01822836 |
| 13 | Cerasibacillus | 0.01357431 | Butyricicoccus | 0.00892026 | Halolactibacillus | 0.01427242 | Halalkalibacillus | 0.01380702 | Flavobacterium | 0.01753026 | Bacteroides | 0.02032269 | Erythromicrobium | 0.01458269 | Tannerella | 0.01799566 |
| 14 | Brachybacterium | 0.01194540 | Parabacteroides | 0.00806701 | Isobaculum | 0.01202296 | Myroides | 0.01326406 | Paenalcaligenes | 0.01613404 | Anaerosporobacter | 0.02016755 | Sinorhizobium | 0.01217810 | Rummeliibacillus | 0.01760782 |
| 15 | Allobacillus | 0.01031648 | Falsibacillus | 0.00736891 | Saccharococcus | 0.01140242 | Paenalcaligenes | 0.01233323 | Azomonas | 0.01504810 | Tannerella | 0.01931431 | **Escherichia_Shigella** | 0.01194542 | Flavonifractor | 0.01605647 |
| 16 | unclassified | 0.00853242 | Solibacillus | 0.00690351 | Streptohalobacillus | 0.01008378 | Falsibacillus | 0.01101459 | Isobaculum | 0.01504810 | Hallella | 0.01838350 | Xylophilus | 0.01109215 | Hallella | 0.01597891 |
| 17 | Yaniella | 0.00767918 | Gemmiger | 0.00550729 | Aeribacillus | 0.00985107 | Nitrincola | 0.01062674 | Faecalibacterium | 0.01427242 | Meniscus | 0.01822836 | Ottowia | 0.00954080 | Oscillibacter | 0.01590134 |
| 18 | Lentibacillus | 0.00760162 | Anaerovirgula | 0.00496432 | Pseudomonas | 0.00938564 | Isobaculum | 0.01008378 | Caryophanon | 0.01380702 | Paraprevotella | 0.01768539 | Methylibium | 0.00930810 | Viridibacillus | 0.01279864 |
| 19 | Nosocomiicoccus | 0.00729135 | Leclercia | 0.00473162 | Amphibacillus | 0.00915297 | Paucisalibacillus | 0.00892026 | Robinsoniella | 0.01078188 | Coprococcus | 0.01644431 | Solibacillus | 0.00915297 | Pseudosphingobacterium | 0.01217810 |
| 20 | Ornithinibacillus | 0.00705864 | Isobaculum | 0.00449891 | Salirhabdus | 0.00798945 | Streptohalobacillus | 0.00860999 | Nitrincola | 0.00946324 | Lachnospiracea_incertae_sedis | 0.01194539 | Pseudacidovorax | 0.00907540 | Meniscus | 0.01039405 |
| 21 | Macrococcus | 0.00690351 | Anaerotruncus | 0.00449891 | Desemzia | 0.00767918 | Salirhabdus | 0.00829972 | Kurthia | 0.00907540 | Caryophanon | 0.01147999 | Xenophilus | 0.00876513 | Oribacterium | 0.00977351 |
| 22 | Desemzia | 0.00667081 | Geosporobacter | 0.00442135 | Melissococcus | 0.00760162 | Atopostipes | 0.00806702 | Wautersiella | 0.00845486 | Dysgonomonas | 0.00985107 | Balneimonas | 0.00860999 | Galbibacter | 0.00884270 |
| 23 | Amphibacillus | 0.00659324 | Sporobacterium | 0.00418865 | Pullulanibacillus | 0.00760162 | Luteimonas | 0.00729135 | Natronobacillus | 0.00806702 | Azomonas | 0.00923053 | Sphingomonas | 0.00860999 | Akkermansia | 0.00845486 |
| 24 | Atopostipes | 0.00628297 | Parasporobacterium | 0.00395594 | unclassified | 0.00752405 | Sporosarcina | 0.00651567 | Persicivirga | 0.00798945 | Isobaculum | 0.00915297 | Xylella | 0.00829972 | Brachybacterium | 0.00845485 |
| 25 | Helcobacillus | 0.00620540 | Flavonifractor | 0.00380081 | Sporosarcina | 0.00667081 | Parapedobacter | 0.00628296 | Lysinibacillus | 0.00798945 | Anaeroglobus | 0.00837729 | TM_genus_incertae_sedis | 0.00775675 | Acetivibrio | 0.00775675 |
| 26 | Salirhabdus | 0.00550729 | Luteolibacter | 0.00380081 | Vulcanibacillus | 0.00659324 | Solibacillus | 0.00535216 | Coprococcus | 0.00791189 | Succiniclasticum | 0.00752405 | Sarcina | 0.00760162 | Paludibacter | 0.00752405 |
| 27 | Jeotgalicoccus | 0.00480919 | Rummeliibacillus | 0.00380081 | Sediminibacillus | 0.00636054 | Oceanobacillus | 0.00504189 | Oribacterium | 0.00736891 | Anaerophaga | 0.00698108 | Luteimonas | 0.00760162 | Prolixibacter | 0.00721378 |
| 28 | Haloactinobacterium | 0.00434378 | Anaerobacter | 0.00372324 | Virgibacillus | 0.00612783 | Filibacter | 0.00496432 | Paralactobacillus | 0.00729135 | Oribacterium | 0.00698107 | Diaphorobacter | 0.00721378 | 5_genus_incertae_sedis | 0.00721378 |
| 29 | Streptohalobacillus | 0.00341297 | Acetanaerobacterium | 0.00372324 | Serpens | 0.00581756 | Psychrobacillus | 0.00403351 | Lachnospiracea_incertae_sedis | 0.00729134 | Lysinibacillus | 0.00682594 | Alicycliphilus | 0.00705864 | Fibrobacter | 0.00698108 |
| 30 | Lacticigenium | 0.00302513 | Tissierella | 0.00349054 | Corynebacterium | 0.00558486 | Allobacillus | 0.00387838 | Tissierella | 0.00705864 | Robinsoniella | 0.00674837 | Croceicoccus | 0.00674837 | Nubsella | 0.00690351 |
| 31 | Thalassobacillus | 0.00302513 | Paludibacter | 0.00341297 | Millisia | 0.00527459 | Cerasibacillus | 0.00380081 | Tannerella | 0.00690351 | Rummeliibacillus | 0.00659324 | Caulobacter | 0.00620540 | Pseudoflavonifractor | 0.00682594 |
| 32 | Sporosarcina | 0.00271486 | Phocaeicola | 0.00333540 | Paenalcaligenes | 0.00504189 | **Escherichia_Shigella** | 0.00333540 | Clostridium_XlVa | 0.00690351 | Clostridium_XIX | 0.00605027 | Salinarimonas | 0.00589513 | Gibbsiella | 0.00682594 |
| 33 | Bhargavaea | 0.00240459 | Clostridium_IV | 0.00325784 | Azomonas | 0.00496432 | Aspromonas | 0.00325784 | Diaphorobacter | 0.00659324 | Megasphaera | 0.00589513 | Piscinibacter | 0.00589513 | Sporobacter | 0.00651567 |
| 34 | Pontibacillus | 0.00232703 | Sporacetigenium | 0.00325784 | Paenisporosarcina | 0.00442135 | Anaerovirgula | 0.00302513 | Rummeliibacillus | 0.00643810 | Lactonifactor | 0.00558486 | Ramlibacter | 0.00535216 | Phascolarctobacterium | 0.00651567 |
| 35 | Bavariicoccus | 0.00224946 | Pseudoflavonifractor | 0.00302513 | Salinibacillus | 0.00434378 | Paralactobacillus | 0.00271486 | Cellulosilyticum | 0.00612783 | Paralactobacillus | 0.00558486 | Stenotrophomonas | 0.00535216 | Saccharofermentans | 0.00589513 |
| 36 | Actinotalea | 0.00209432 | Alkalibaculum | 0.00294757 | Bhargavaea | 0.00403351 | Eionea | 0.00271486 | Sporacetigenium | 0.00527459 | Nubsella | 0.00550729 | Pimelobacter | 0.00527459 | Yaniella | 0.00558486 |
| 37 | Paraliobacillus | 0.00193919 | Petrimonas | 0.00279243 | Paraliobacillus | 0.00403351 | Sporacetigenium | 0.00255973 | Lachnobacterium | 0.00519702 | Alkanindiges | 0.00542972 | Aquincola | 0.00527459 | Psychrobacillus | 0.00542973 |
| 38 | Isobaculum | 0.00170649 | Serpens | 0.00271486 | **Escherichia_Shigella** | 0.00403351 | Bavariicoccus | 0.00240459 | Bacteroides | 0.00511946 | Wautersiella | 0.00519702 | Brachymonas | 0.00527459 | Parasporobacterium | 0.00542973 |
| 39 | Halolactibacillus | 0.00170649 | Bacillus | 0.00263730 | Abiotrophia | 0.00395594 | Proteiniphilum | 0.00232703 | Succiniclasticum | 0.00511946 | Gallicola | 0.00457648 | Lysinibacillus | 0.00511946 | Paraprevotella | 0.00511945 |
| **40** | **Escherichia_Shigella** | 0.00170649 | Ruminococcus | 0.00263730 | Halothermothrix | 0.00372324 | Modicisalibacter | 0.00232703 | Melissococcus | 0.00504189 | Flavobacterium | 0.00457648 | Azorhizophilus | 0.00504189 | Salinicoccus | 0.00473162 |
| 41 | Granulicatella | 0.00170649 | Bacteroides | 0.00255973 | Filibacter | 0.00364567 | Halolactibacillus | 0.00224946 | Millisia | 0.00496432 | Millisia | 0.00442135 | Clostridium_sensu_stricto | 0.00504189 | Clostridium_XlVa | 0.00473161 |
| 42 | Murinocardiopsis | 0.00170649 | Pseudoxanthomonas | 0.00232703 | Solibacillus | 0.00356811 | Paenisporosarcina | 0.00217189 | Alkanindiges | 0.00496432 | **Escherichia_Shigella** | 0.00442135 | Rudaea | 0.00496432 | Papillibacter | 0.00465405 |
| 43 | Brevibacterium | 0.00170648 | Stakelama | 0.00232703 | Jeotgalibacillus | 0.00356811 | Rummeliibacillus | 0.00209432 | Acholeplasma | 0.00496432 | Paenalcaligenes | 0.00426621 | Crabtreella | 0.00480919 | Bhargavaea | 0.00449892 |
| 44 | Rummeliibacillus | 0.00162892 | Blastomonas | 0.00232703 | Nosocomiicoccus | 0.00341297 | Nubsella | 0.00209432 | Planococcaceae_incertae_sedis | 0.00488675 | Pseudoflavonifractor | 0.00411108 | Asticcacaulis | 0.00473162 | Verrucomicrobium | 0.00418865 |
| 45 | Saccharococcus | 0.00155135 | Aspromonas | 0.00217189 | Macrococcus | 0.00325784 | Olleya | 0.00209432 | **Escherichia_Shigella** | 0.00480919 | Sporacetigenium | 0.00364567 | Prosthecobacter | 0.00473162 | Cosenzaea | 0.00411108 |
| 46 | Pisciglobus | 0.00155135 | Sporanaerobacter | 0.00217189 | Lentibacillus | 0.00310270 | Aidingimonas | 0.00201676 | Aspromonas | 0.00465405 | Parasutterella | 0.00364567 | Nubsella | 0.00465405 | Planococcaceae_incertae_sedis | 0.00403351 |
| 47 | Geomicrobium | 0.00116351 | Saccharofermentans | 0.00217189 | Pontibacillus | 0.00302513 | Ignavigranum | 0.00193919 | Sporobacterium | 0.00457648 | Flavonifractor | 0.00341297 | Hylemonella | 0.00457648 | Perlucidibaca | 0.00387838 |
| 48 | Aeribacillus | 0.00108595 | Erythromicrobium | 0.00209432 | Filobacillus | 0.00271486 | Bhargavaea | 0.00178405 | Psychrobacillus | 0.00449892 | Barnesiella | 0.00341297 | Tannerella | 0.00449892 | Lactonifactor | 0.00372324 |
| 49 | Atopobacter | 0.00108595 | Aeribacillus | 0.00201676 | Pisciglobus | 0.00240459 | Soehngenia | 0.00178405 | Pseudoflavonifractor | 0.00449892 | Cellulosilyticum | 0.00333540 | Bhargavaea | 0.00442135 | Nosocomiicoccus | 0.00364567 |
| 50 | Salinibacillus | 0.00100838 | Cosenzaea | 0.00201676 | Thermoactinomyces | 0.00240459 | Flaviramulus | 0.00178405 | Gilvimarinus | 0.00449892 | Butyricicoccus | 0.00333540 | Thermomonas | 0.00434378 | Phocaeicola | 0.00356811 |
