# Supplementary figures and images for: Tracking Enterobacteria, microbiomes, and antibiotic resistance genes from waste to soil with repeated compost applications
Source: PLoS One. 2025 Aug 13;20(8):e0329200. doi: 10.1371/journal.pone.0329200 (PMC12349694; doi:10.1371/journal.pone.0329200)

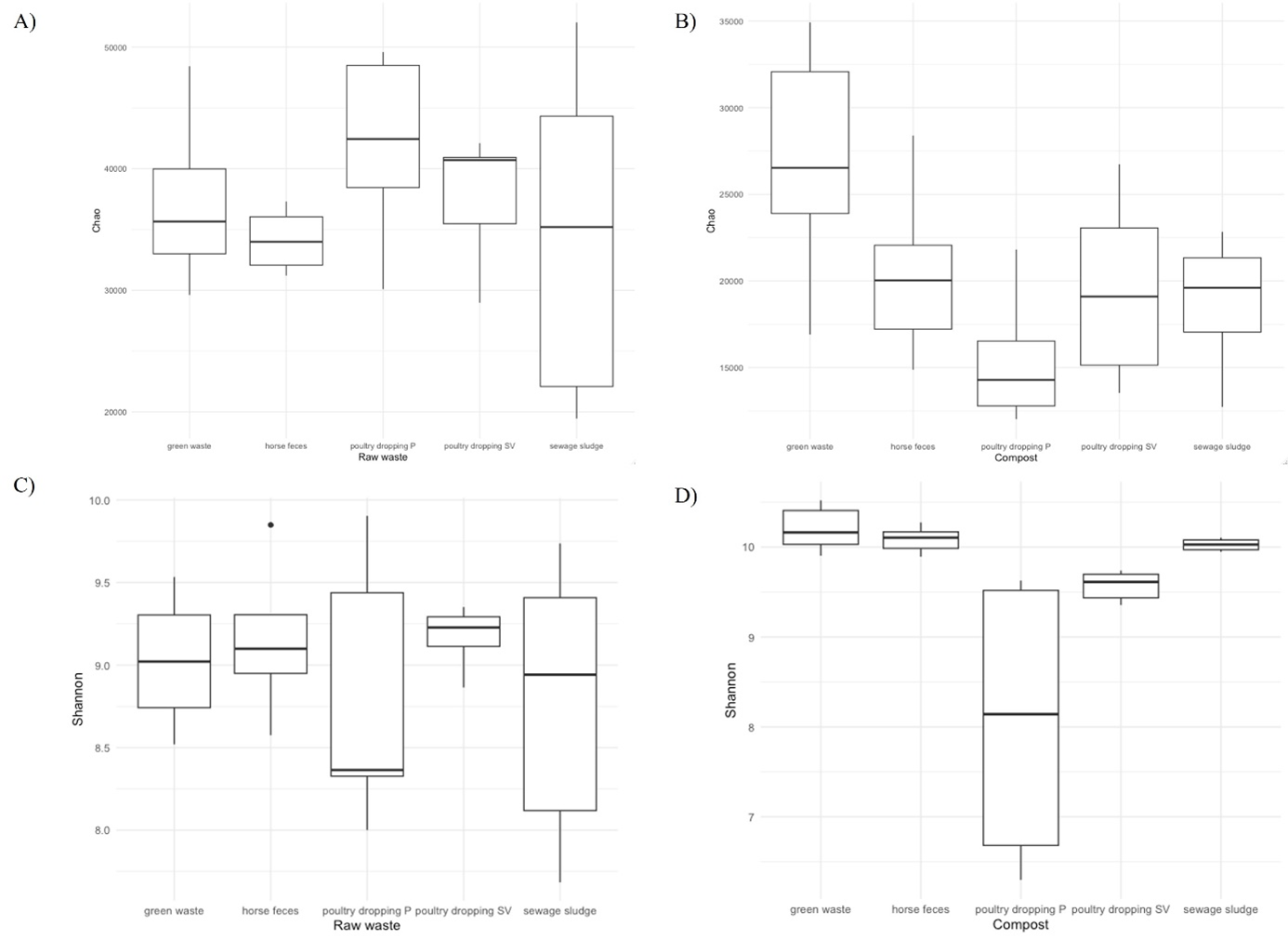

Supplement: S1 Fig — Box-plots of the Chao (A and B) and Shannon indexes (C and D) for the raw waste and their derived-composts. (TIF) [file pone.0329200.s004.tif]

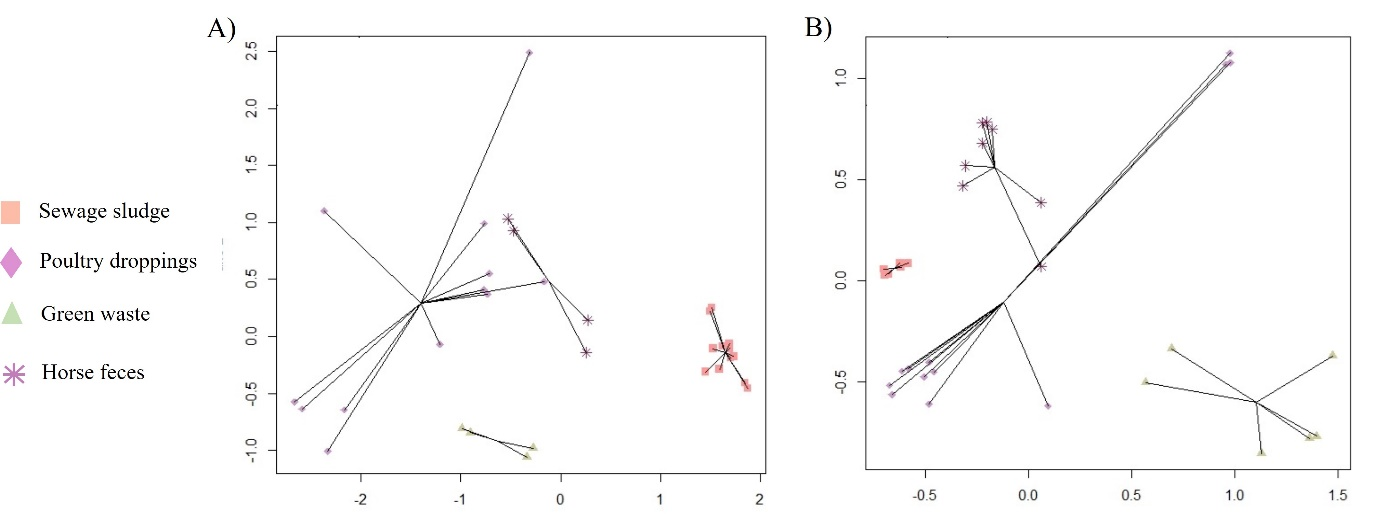

Supplement: S2 Fig — Bray–Curtis similarity coefficients were calculated from relative OTU abundances of bacterial communities and plotted on a nonmetric multidimensional scaling (NMDS) graph. (TIF) [file pone.0329200.s005.tif]

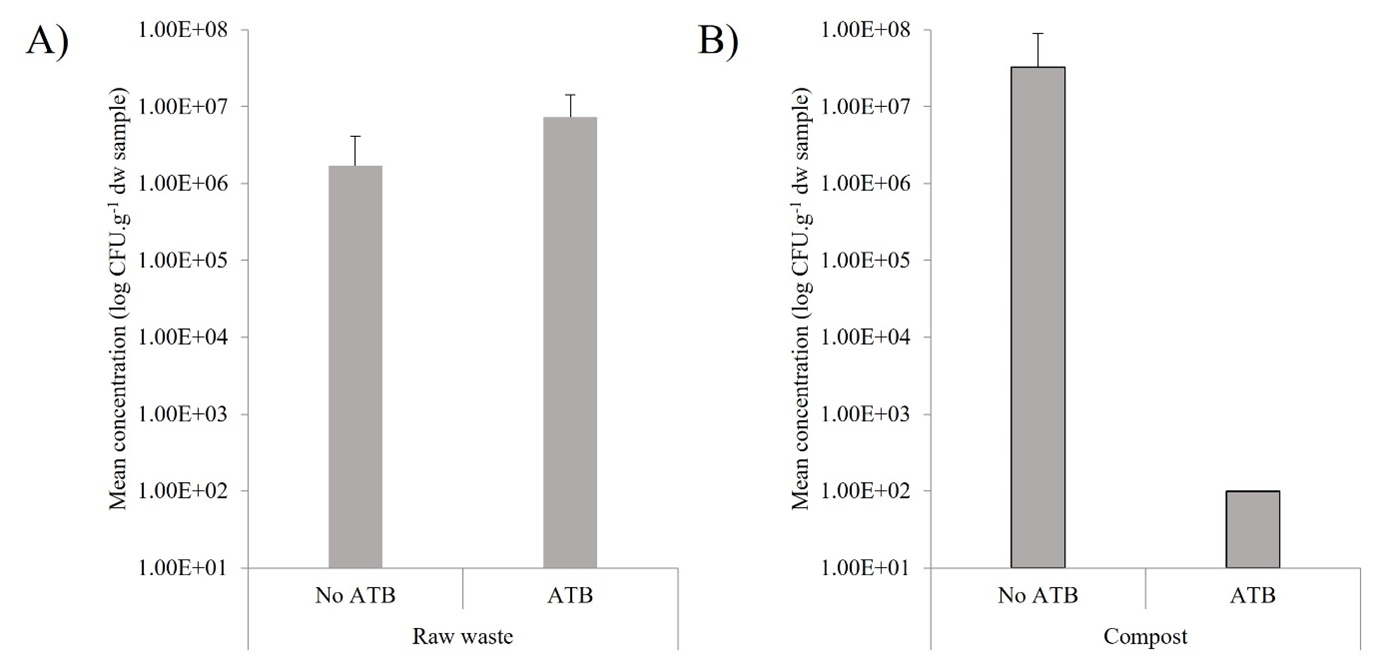

Supplement: S3 Fig — A) Raw waste: Untreated horse feces (n = 2), Antibiotic-treated horse feces (n = 3) B) Composts: Untreated horse feces (n = 3), Antibiotic-treated horse feces (n = 1). ATB: antibiotic. CFU, colony forming unit. Mean concentrations are shown in CFU per gram of dry weight sample (CFU.g-1 sdw). Error bars represent the standard deviation of at least two independent experiments. (TIF) [file pone.0329200.s006.tif]

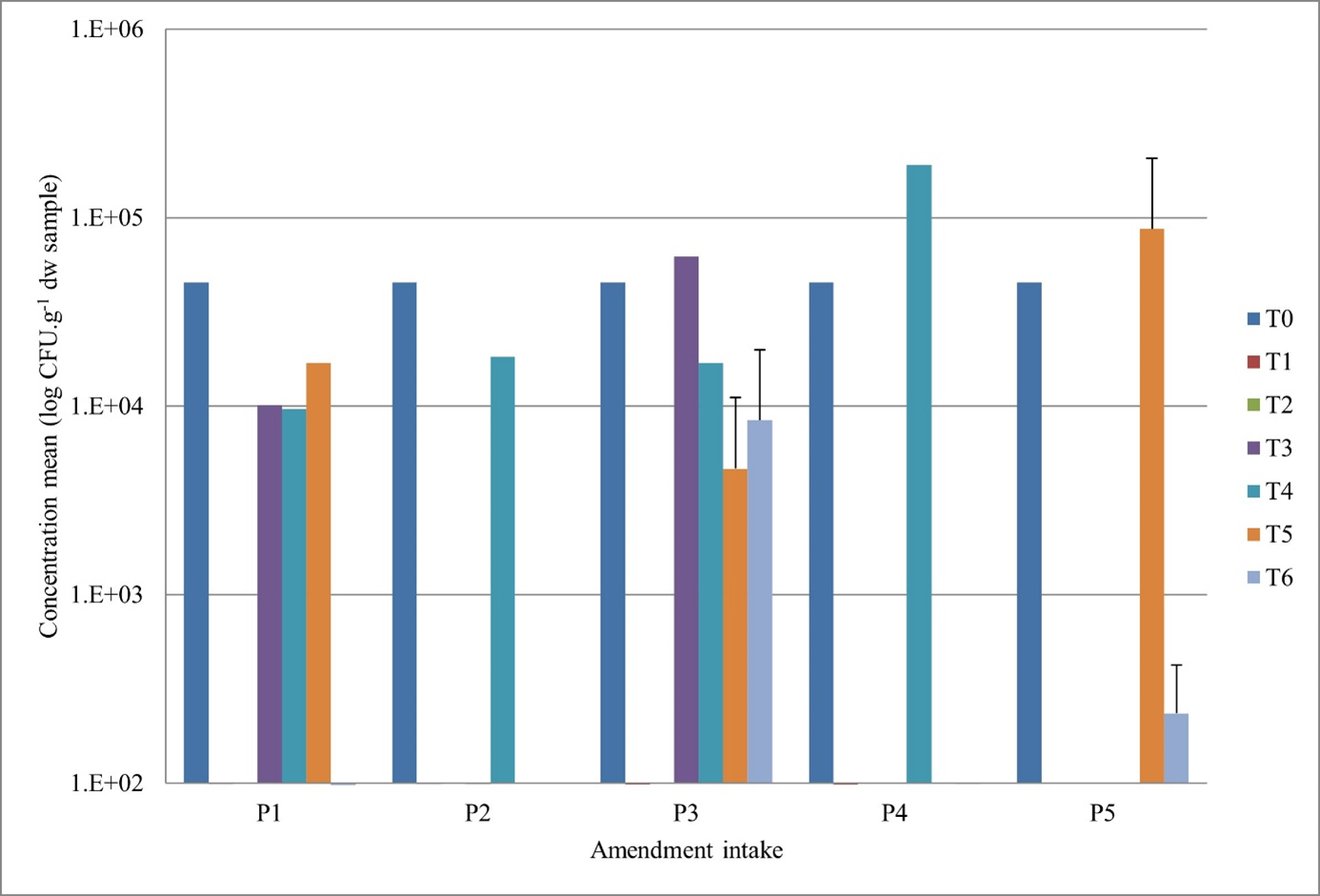

Supplement: S4 Fig — Six plots (P1 to P6) were loaded at different times session during the crop production periode (T0 to T6), according to the following sheme; P1: no raw waste/ green waste/ green waste – slots 1 and 2, P2: horse feces/ horse feces/ poultry dropping – slots 3 and 4, P3: poultry droppings/ horse feces/ poultry droppings – slots 5 and 6, P4: poultry droppings/ poultry droppings/ poultry droppings – slots 7 and 8, P5: no raw waste/ sewage sludge – slots 9 and 10; and time: T0: before the 1st session, T1: after the first raw waste application in the middle of the 1st session, T2: after plant harvest and before the second application at the end of the 1st session, T3: after the second raw waste application in the middle of the 2nd session, T4: after plant harvest and before the third application at the end of the 2nd session, T5: after the third raw waste application in the middle of the 3rd session, T6: after plant harvest at the end of the 3rd session. (TIF) [file pone.0329200.s007.tif]

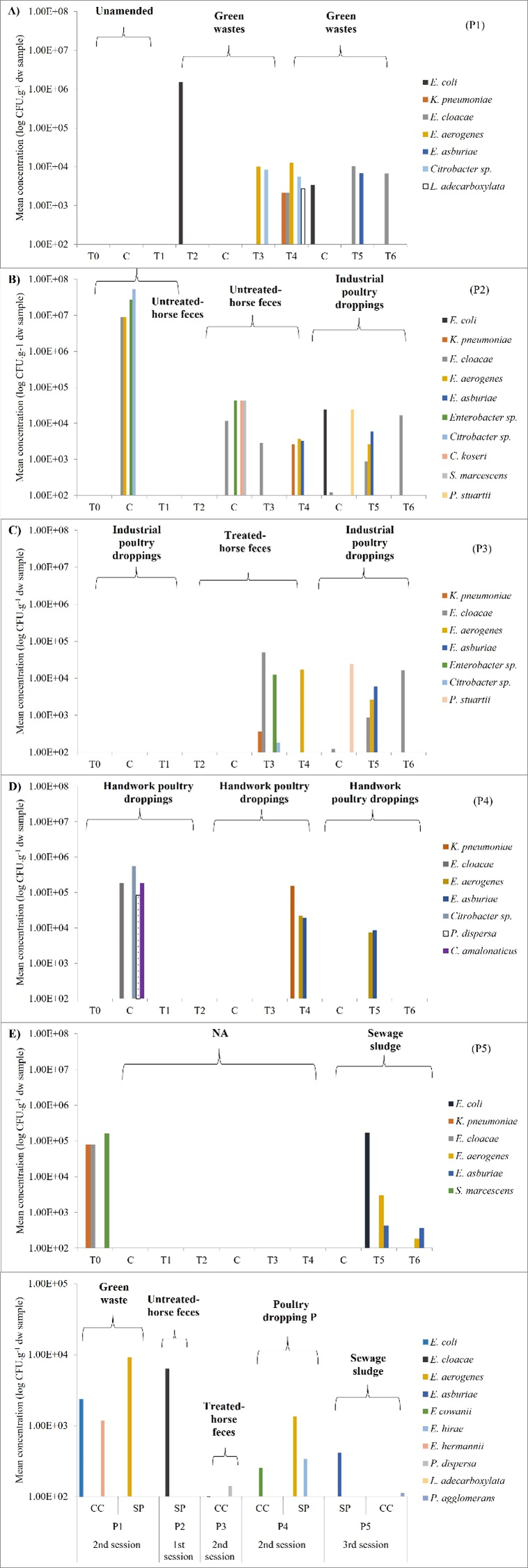

Supplement: S5 Fig — A) P1 successive application of no raw waste/ green waste/ green waste, B) P2 successive application of horse feces/ horse feces/ poultry dropping, C) P3 successive application of: poultry droppings/ horse feces/ poultry droppings, D) P4 successive application of poultry droppings/ poultry droppings/ poultry droppings, E) P5 successive application of no raw waste/ sewage sludge, F) Mean concentration of resistant enterobacteria by species in vegetables (CC and SP) after successive applications of amendments (P1 to P5). T0: before the 1st session, T1: after the first raw waste application in the middle of the 1st session, T2: after plant harvest and before the second application at the end of the 1st session, T3: after the second raw waste application in the middle of the 2nd session, T4: after plant harvest and before the third application at the end of the 2nd session, T5: after the third raw waste application in the middle of the 3rd session, T6: after plant harvest at the end of the 3rd session. No plot indicates that no resistant enterobacteria were detected, except for slots 9–10 (P5) where sewage sludge compost was applied only in the 3rd session. CFU: colony forming unit, C: compost, CC, cucumber; SP, sweet potatoes, NA: not applicable. (TIF) [file pone.0329200.s008.tif]
