# Supplementary material for: Correction: Tracking Enterobacteria, microbiomes, and antibiotic resistance genes from waste to soil with repeated compost applications
Source: PLoS One. 2025 Nov 18;20(11):e0337209. doi: 10.1371/journal.pone.0337209 (PMC12626308; doi:10.1371/journal.pone.0337209)
Supplement: S1 File — These include detailed descriptions of materials and methods (such as plot characteristics, soil properties, experimental design, composting conditions, application rates, sampling procedures, bacterial analysis protocols, and molecular methods including DNA extraction, qPCR conditions, and primer sequences), as well as supplementary tables (S1–S3) and figures (S1–S5) illustrating the results. (PDF) [file pone.0337209.s001.pdf]

Materials and methods

Study area

Field experiments were carried out on an experimental plot of 150 m^2, located at Petit-Bourg, in an agricultural area in the north of Basse-Terre, Guadeloupe, French West Indies, (latitude 16°11’52’’N, longitude 61°36’33’’W), from October 2015 to February 2017. The soil was characterized by a pH of 7.9, a cation exchange capacity of 29 $cmol.kg^{-1}$, a sand:silt:clay ratio (%) of 15:23:62, and an organic matter content of 54.3 $g.kg^{-1}$. The field plot was left fallow for 5 years.

Raw waste and compost

Amendments were made to different raw waste composts, including those derived from animal sources (horse feces, poultry droppings) and from domestic and urban activities (sewage sludge). The raw waste materials were mixed with green waste for the purpose of producing compost. The production of compost from green waste was also examined. To ensure optimal composting conditions, a minimum of $1m^3$ of raw waste was required for all composts under study. The hand-made compost, produced by a farmer Pitaya (P), was maintained for a period of 4 months (poultry droppings, horse feces) whereas the industrial compost, made by the Sita Verde (SV) company (green waste, sewage sludge, poultry droppings) was kept for a minimum of one month). The horse feces treated with antibiotics required a longer period of composting (10 months) due to the lack of sufficient feces to reach 1 m^3. Therefore, only one such compost was produced. Temperature was monitored regularly, and the compost was turned weekly to maintain aerobic conditions and ensure a consistent temperature throughout the pile. The temperature in the swath was controlled to be above 80°C. The applications of the composts used per slot and session are detailed in Fig 1.

Fig 1. Amendment design. Experimental plot, amendment intake and temporal scale were designed as slots: P1: no raw waste/ green waste/ green waste - slots 1 and 2, P2: horse feces/ horse feces/ poultry dropping - slots 3 and 4, P3: poultry droppings/ horse feces/poultry droppings - slots 5 and 6, P4: poultry droppings/ poultry droppings/ poultry droppings - slots 7 and 8, P5: no raw waste/ sewage sludge - slots 9 and 10; and time: T0: before the 1st session, T1: after the first raw waste application in the middle of the 1st session, T2: after plant harvest and before the second application at the end of the 1st session, T3: after the second raw waste application in the middle of the 2nd session, T4: after plant harvest and before the third application at the end of the 2nd session, T5: after the third raw waste application in the middle of the 3rd session, T6: after plant harvest at the end of the 3rd session.

Experimental design

The experimental field plot was subdivided into 10 identical slots of 15 m by 1 m. The study was carried out over two sessions of vegetable crops; cucumbers (*Curcumis sativus*) and sweet potatoes (*Ipomoae batatas*) on amended plots. Two plots were subjected to the same amendment during the same session. Two plots of unamended soil were used as a control (Fig 1). The composts were applied at a rate of approximately 0.01 m^3 of compost per m^2 of soil, which is the average quantity typically employed by farmers for their crops. The study comprised three crop sessions, with an average duration of 5 months, from the preparation of the compost to the harvest. The first session (S1) lasted from October 2015 to February 2016, the second session (S2) from March 2016 to July 2016, and the third session (S3) from August 2016 to February 2017. A one-month period was allowed to elapse between each session, during which time the field plot was free of vegetal cover. Sewage sludge was applied only during the 3rd session, in a slot distant from 25 m away. A soil sample of this slot was taken as a control

before the application of compost.

Sampling

At each session, samples from raw waste, and mature compost (1kg) were randomly selected. Before the application of any composts, 1 month following the application of compost, and 1 month following the harvest, 10 points within the 1 kg sample were selected and pooled. Unamended control soil samples were taken before the beginning of the study, in September 2015. Subsequently, soil samples were sieved at 2.8 mm and 1.85 mm. Cucumbers and sweet potatoes have the same production cycle. They were randomly collected at the same time of each session when the crops were mature and ready for consumption. The sampling periods were as follows: S1 (February 2016), S2 (July 2016) and S3 (February 2017). The samples were placed in bags and transported to the laboratory in a cooler with ice packs.

Bacterial isolation, identification and count

The preparation of compost, soil and vegetable suspensions for viable plate count was conducted by mixing 50 g of the sample in 500 ml of NaCl at 0.25 % (Sigma-Aldrich, Missouri, USA). Concerning raw waste, 30 g were mixed in 200 ml peptone water (Biokar Diagnostics, Allonne, France). Subsequently, the homogenized samples were aseptically diluted 10-fold, in the same solution and plated on 2 agar media. Lactose-triphenyl tetrazolium chloride-agar with Tergitol-7 (TTC) (Biokar Diagnostics, Allonne, France) was used for the cultivation and enumeration of Enterobacteria. TTC was used with and without an antibacterial agent for each sample. The antibiotics were tested at concentrations that select for resistant bacteria. Selective TTC media were supplemented with ampicillin (4 mg/L), cefotaxime (2 mg/L), imipenem (4 mg/L), or ciprofloxacin (1 mg/L). Enterobacteria enumerated on these selective media were classified as resistant Enterobacteria. Plate count agar (PCA) media (Laboratoires Humeau, La

Chapelle-sur-Erdre, France) was used to enumerate total microflora. The incubations were conducted for a period of between 24h and 72h at 37°C.

For each sample, an Enterobacterial count was performed by enumerating Colony Forming Unit (CFU) on TTC (yellow or pink-red colonies, oxidase negative, lactose positive) with and without ampicillin. A maximum of 10 presumptive Enterobacterial colonies were randomly selected and identified by matrix-assisted laser desorption/ionization time-of-flight mass spectrometry (MALDI TOF) on an Axima performance spectrometer (Shimadzu Corp, Osaka, Japan). The frequency of resistant Enterobacteria by species was estimated by calculating the ratio of bacteria per species on TTC with and without antimicrobial agent.

Antimicrobial susceptibility testing

The antimicrobial susceptibility of all Enterobacteria strains isolated from raw waste, composts and vegetable samples was assessed using the disk diffusion technique on Mueller-Hinton agar, as recommended by EUCAST 2017 (http://www.eucast.org). The following antibiotics were tested: ampicillin (10 µg), amoxicillin-clavulanic acid (20 µg-10 µg), piperacillin tazobactam (30 µg-6 µg), ticarcillin (75µg), cephalexin (30µg), cefoxitin (30 µg), cefotaxime (5 µg), ceftazidime (10 µg), ertapenem (10 µg), aztreonam (30 µg), gentamicin (10 µg), amikacin (30 µg), nalidixic acid (30 µg), ciprofloxacin (5 µg), tigecycline (15 µg), and trimethoprim-sulfamethoxazole (1.25 µg-23.75 µg).

The presence of ESBL producing Enterobacteria was confirmed through the combined disk diffusion test, utilizing cefotaxime and ceftazidime, with and without clavulanic acid. The inhibition zones were measured using the Adagio™ automated system (Bio-Rad, Marnes-La-Coquette, France). Enterobacteria strains were classified as susceptible, intermediate, or resistant according to the guidelines of EUCAST. *E. coli* ATCC 25922 was used as the control strain.

DNA extraction

Genomic bacterial DNA was extracted from a single colony using an InstaGene™ Matrix kit following the manufacturer's instructions (Biorad, California, USA). Total DNA from 0.34 g of dry raw waste, compost and soil samples was extracted in triplicate using the NucleoSpin® soil kit (Macherey Nagel, Hoerdt, France) following the manufacturer's instructions. The quality of extracted DNA was analysed using 0.8% agarose gel electrophoresis. The DNA concentration was further estimated using a Nanodrop® ND-1000 spectrophotometer (Labtech International, Paris, France) at a wavelength of 260 nm.

ARGs molecular characterization

The presence of extended spectrum β-lactamase, cephalosporinase encoding genes, sulfonamides and quinolone plasmidic resistances were screened by PCR in all Enterobacteria isolates that had been phenotypically characterized as resistant by antimicrobial susceptibility testing. The *Escherichia coli* strains designated as positive control were provided by Pr. G. Arlet (Hôpital Tenon, Paris, France). The primers used were previously described in the literatured. A $bla_{\text{CTX-M}}$ multiplex PCR including phylogenetic groups 1, 2 and 9 was performed (1). The presence of the AmpC β-lactamase genes, which are frequently found in Enterobacteria, was tested by multiplex PCR using the MOX (for detecting $bla_{\text{MOX-1, -2}}$, $bla_{\text{CMY-1, -8 to -11}}$ genes), CIT (for detecting $bla_{\text{LAT-1 to -4}}$, $bla_{\text{CMY-2 to -7}}$, $bla_{\text{BIL-1}}$ genes), DHA (for detecting $bla_{\text{DHA-1, -2}}$ genes), ACC (for detecting bla_{AAC} gene), EBC (for detecting $bla_{\text{MIR-1}}$, $bla_{\text{ACT-1}}$ genes and FOX (for detecting $bla_{\text{FOX-1 to -5b}}$ genes) primers as previously described (2). A duplex PCR was used to characterize *qnr*B and *qnr*S genes (3). The screening of bla_{TEM}, bla_{SHV} and *sul* was performed by simplex PCR (4–6). The amplified PCR products were sequenced by Eurofins (Ivry sur Seine, France), and compared with known resistance gene sequences hosted in

GenBank database by multiple sequence alignment using the BLAST program for further characterization.

Quantification of gene target copies using qPCR and ddPCR

The genes encoding 16S rRNA and antibiotic resistance were quantified in 28 raw waste samples comprising 12 poultry droppings (6 from P and 6 from SV), 4 horse feces, 12 sewage sludges and 4 green waste samples. Additionally, the same genes were quantified in their derived-composts, which included 12 poultry droppings (6 from P and 6 from SV), 8 horse feces, 6 sewage sludges, and 6 green waste samples. Real-time quantitative PCR (qPCR) and digital droplet PCR (ddPCR) were used for quantification. qPCR was applied on DNA extracts to quantify 16S rRNA gene copies and sulfonamide resistance genes (*sul*1, *sul*2). ddPCR was used to quantify β-lactam resistance genes ($bla_{\text{CTXM-1}}$, $bla_{\text{CTXM-9}}$, $bla_{\text{IMP-1,2,3}}$), fluoroquinolone resistance genes (*qnr*A, *qnr*B) as well as class I and class II integrase genes (*intI*1 and *intI*2). The use of ddPCR was justified by the evidence that it is more sensitive and accurate for low copy numbers and less sensitive to inhibitors when used to quantify genes in soil and organic residues (7).

A summary of the primers and probes used in the present study can be found in S2 Table. The primers and probes were synthesized by Invitrogen (Cergy Pontoise, France) and Metabion (Munich, Germany), respectively. Quantitative PCR was performed using a Bio-Rad CFX96 real-time PCR instrument with Bio-Rad CFX Manager software, version 3.0. (Bio-Rad). ddPCR was performed on the QX100™ Droplet Digital™ PCR system (Bio-Rad). Quantitative PCR reaction mixtures were prepared with the Sso Advanced™ Universal Probe Supermix. A volume of 5 µl of template DNA (equivalent to 1 and 10 ng of DNA), combined with 0.5 µl of 500 ng of T4 bacteriophage gene 32 protein (MP Biomedicals), was added to a final volume of 25 µl, which was composed of deionized water. Negative controls lacking template DNA were

conducted in triplicate. Each reaction was duplicated with both DNA template concentrations, using the following cycle conditions: 1 cycle at 95°C for 10 min followed by 40 cycles of 95°C for 10 s and annealing temperature for 10 or 20 s. With regards to ddPCR the mix reactions were performed with the ddPCR™ Supermix for Probes with no dUTP kit (Bio-Rad) using 10 and 50 ng of DNA *per* reaction. The reactions were set up at a final volume of 20 µl at 500 nM for each primer and 200 nM for the probe, in accordance with the manufacturer's instructions.

Metabarcoding analysis

The V3-V4 regions (forward: TACGGRAGGCAGCAG and reverse: TAGGATTAGATACCCTGGTA) of 16S rRNA genes from DNA extracts were sequenced at the DTAMB/ Biofidal platform (University of Lyon 1, France) using an Illumina MiSeq platform and paired-end reads. The metabarcoding analysis was carried out following the Mothur MiSeq standard operating procedure (SOP) pipeline v1.35.1 (http://www.mothur.org/wiki/MiSeq_SOP) developed by the Schloss laboratory (Department of Microbiology & Immunology, University of Michigan, USA) (8,9). Briefly, the contigs of paired-end read 1 and read 2 were constructed using the *make.contigs* command and sequences containing ambiguous bases, as well as those exceeding 550 bp or falling below 400 bp were removed using the *screen.seqs* command. The unique sequences were obtained using the *unique.seqs* command, and the *align.seqs* command was then used to align the data with Mothur's SILVA bacterial 16S reference alignment v123 (https://www.arb-silva.de) (10). Following the screening of the unique sequences, the remaining reads were pre-clustered to denoise the sequences within each sample. Additionally, chimeric sequences were removed using the *chimeric.uchime* command (http://drive5.com/uchime) (11). Subsequently, each sequence was classified against the RDP 16S rRNA training set v9 using a naïve Bayesian classifier, with an 80% confidence level (12). The sequences were regrouped into operational

taxonomic units (OTUs) with a 3% divergence threshold using the *cluster.split* command (http://www.mothur.org/wiki/Cluster.split). Additionally, the Chao1 richness estimator and the Np Shannon diversity were calculated.

References

1. Dallenne C, da Costa A, Decré D, Favier C, Arlet G. Development of a set of multiplex PCR assays for the detection of genes encoding important β-lactamases in Enterobacteriaceae. Journal of Antimicrobial Chemotherapy. 2010;65(3):490–5. DOI: 10.1093/jac/dkp498

2. Pérez-Pérez FJ, Hanson ND. Detection of plasmid-mediated AmpC beta-lactamase genes in clinical isolates by using multiplex PCR. J Clin Microbiol. 2002;40(6):2153–62. DOI: 10.1128/JCM.40.6.2153

3. Guessennd N, Bremont S, Gbonon V, Kacou-NDouba A, Ekaza E, Lambert T, et al. Résistance aux quinolones de type qnr chez les entérobactéries productrices de bêta-lactamases à spectre élargi à Abidjan en Côte d'Ivoire. Pathologie Biologie. 2008;56(7–8):439–46. DOI: 10.1016/j.patbio.2008.07.025

4. Eckert C, Gautier V, Saladin-Allard M, Hidri N, Verdet C, Ould-Hocine Z, et al. Dissemination of CTX-M-type beta-lactamases among clinical isolates of *Enterobacteriaceae* in Paris, France. Antimicrob Agents Chemother. 2004/03/30. 2004;48(4):1249–55. DOI: 10.1128/aac.48.4.1249-1255.2004

5. Lanz R, Kuhnert P, Boerlin P. Antimicrobial resistance and resistance gene determinants in clinical *Escherichia coli* from different animal species in Switzerland. Vet Microbiol. 2002/11/21. 2003;91(1):73–84. DOI: S0378113502002638 [pii]

6. Arlet G, Rouveau M, Philippon A. Substitution of alanine for aspartate at position 179 in the SHV-6 extended-spectrum β-lactamase. FEMS Microbiol Lett. 1997 Jul;152(1):163–7. DOI: 10.1016/S0378-1097(97)00196-1

7. Cavé L, Brothier E, Abrouk D, Bouda PS, Hien E, Nazaret S. Efficiency and sensitivity of the digital droplet PCR for the quantification of antibiotic resistance genes in soils and organic residues. Appl Microbiol Biotechnol. 2016 Dec;100(24):10597–608. DOI: 10.1007/s00253-016-7950-5

8. Schloss PD, Westcott SL, Ryabin T, Hall JR, Hartmann M, Hollister EB, et al. Introducing mothur: open-source, platform-independent, community-supported software for describing and comparing microbial communities. Appl Environ Microbiol. 2009 Dec;75(23):7537–41. DOI: 10.1128/AEM.01541-09

9. Kozich JJ, Westcott SL, Baxter NT, Highlander SK, Schloss PD. Development of a dual-index sequencing strategy and curation pipeline for analyzing amplicon sequence data on the MiSeq Illumina sequencing platform. Appl Environ Microbiol. 2013 Sep;79(17):5112–20. DOI: 10.1128/AEM.01043-13

10. Quast C, Pruesse E, Yilmaz P, Gerken J, Schweer T, Yarza P, et al. The SILVA ribosomal RNA gene database project: improved data processing and web-based tools. Nucleic Acids Res. 2013 Jan;41(Database issue):D590-6. DOI: 10.1093/nar/gks1219

11. Edgar RC, Haas BJ, Clemente JC, Quince C, Knight R. UCHIME improves sensitivity and speed of chimera detection. Bioinformatics. 2011 Aug;27(16):2194–200. DOI: 10.1093/bioinformatics/btr381

12. Wang Q, Garrity GM, Tiedje JM, Cole JR. Naive Bayesian classifier for rapid assignment of rRNA sequences into the new bacterial taxonomy. Appl Environ Microbiol. 2007 Aug;73(16):5261–7. DOI: 10.1128/AEM.00062-07
